# Association Between Social Determinants of Health and Cardiovascular Risk in Pregnant Women, United States

**DOI:** 10.5888/pcd23.250298

**Published:** 2026-02-26

**Authors:** Shazia Shaukat, Jennifer Horney, Xiaopeng Ji, Christine Hoch, Lauren Covington

**Affiliations:** 1 Department of Epidemiology, University of Delaware, Newark, Delaware; 2 School of Nursing, University of Delaware, Newark, Delaware

## Abstract

**Introduction:**

Poor cardiovascular health (CVH) accounts for one-third of pregnancy-related deaths in the US. Social determinants of health (SDOH) — conditions in which people are born, grow, work, live, and age — can negatively influence maternal CVH. Little is known about potential negative effects of SDOH on CVH during the perinatal period. This study aimed to identify associations between SDOH with CVH in pregnant women.

**Methods:**

We conducted a cross-sectional analysis of women who participated in the Pregnancy Risk Assessment and Monitoring System (PRAMS) survey between 2016 and 2020. We created a composite score for CVH status based on the presence of hypertension, diabetes, smoking, and obesity. SDOH variables were maternal education level, marital status, rural residency, work status, poverty level, insurance status, food security, history of breastfeeding, birth order of the child, history of abuse, and depression history. Logistic regression accounting for complex survey data evaluated the association of SDOH with poor CVH risk by using adjusted odds ratios (AORs) and 95% CIs.

**Results:**

The final sample was 205,513 pregnant women (57.2% non-Hispanic white, 15.1% non-Hispanic Black, 18.3% Hispanic), of whom 53.8% were classified as having poor CVH. Disadvantaged SDOH were associated with poor CVH: rural residence (OR = 1.15), food insecurity (OR = 1.36), abuse history (OR = 1.43), depression history (OR = 1.52), living at or below the federal poverty line (OR = 1.23), single marital status (OR = 1.38), less than high school diploma (OR = 1.13) and having government insurance (OR = 1.31) (all *P* < .05).

**Conclusions:**

Having disadvantaged SDOH was associated with poor CVH during pregnancy.

**Summary**

**What is already known about this topic?**

Pregnancy is a unique window to study women seeking routine health care and to assess the potential for intervention to address health disparities.

**What is added by this report?**

In a sample of pregnant women, we found a significant association between adverse cardiovascular health outcomes and disadvantaged social determinants of health (SDOH) among all races and ethnicities, with a pronounced increase in odds of adverse outcomes among White women.

**What are the implications for public health practice?**

Addressing SDOH necessitates social and environmental policy changes that inform health interventions. Health care providers should consider these factors in their cardiovascular health care strategies and advocate for policy changes that can support development of health behavior interventions.

## Introduction

Complications related to cardiovascular disease account for more than one-third of pregnancy-related deaths in the US and are a significant cause of maternal morbidity (1). Poor cardiovascular health (CVH) of women during the perinatal period increases rates of morbidity and mortality for both the mother and her child across the life course (2,3), and the risks are higher for minority racial and ethnic populations (4–6). CVH is assessed by using behavioral and lifestyle indicators. The American Heart Association identifies Life’s Essential 8: eat better, get active, stop smoking, and get healthy sleep, as modifiable behaviors; and lose weight, manage blood pressure, control cholesterol, and reduce blood glucose as manageable health factors that can improve CVH (7). These factors are intergenerational, such that improving them in mothers will also have lifelong positive implications for the child (8).

Social determinants of health (SDOH) are the conditions in which people are born, grow, live, work, and age, as well as the wider set of forces and systems shaping the conditions of daily life (9). More recently, the understanding of SDOH has evolved and broadened to encompass a range of social and environmental factors that influence health outcomes. The Kaiser Foundation Model (10) and Healthy People 2030 (11) advocate for a comprehensive approach to improving population-level health outcomes and elimination of disparities and inequities through addressing SDOH. As such, consideration of SDOH must be central to public health interventions aiming to improve CVH.

The Healthy People 2030 framework uses 5 domains focused on 1) economic stability, 2) education quality, 3) health care quality and access, and 4) neighborhood and 5) community contexts to assess SDOH (11). The World Health Organization characterizes determinants of health along multiple levels including personal characteristics and behaviors, physical environment, and social–environmental conditions (12). An ecological, multilevel modeling approach can be used to assess health issues in diverse populations and produce data to guide actions at individual, interpersonal, and community levels (13). Accordingly, this research takes an applied approach inspired by ecological modeling and categorizes SDOH based on individual, interpersonal, and community levels.

Pregnancy may be an ideal time in the life course to address CVH, given that preconception counseling and continuous individualized antenatal care occur during pregnancy. However, many women do not have access to such care due to disadvantaged SDOH that intersect at the individual (eg, health literacy), interpersonal (eg, lack of social support), community (eg, unsafe neighborhood), and societal and policy (eg, access to care) levels. Cumulative risks secondary to SDOH are associated with poor CVH in pregnant women (14). Although a few studies have examined the interplay between SDOH and health outcomes for pregnant women (14,15), these studies are retrospective and not connected to vital records that can validate responses. Furthermore, the findings of these studies were not conducted in a manner that could address individual, interpersonal, community, and policy dimensions of SDOH.

Being a member of a racial or ethnic minority group is associated with increased risk of morbidity, mortality (4), and poor CVH (5) in pregnant women. Racial and ethnic minority groups often experience varying degrees of SDOH disadvantage, increasing their risk for poor CVH (16). However, the extent to which the association between SDOH and CVH in pregnant women varies based on maternal race and ethnicity is not known. Identifying which racial and ethnic minority groups are most at risk for negative effects of SDOH on CVH will allow for the creation of individualized health interventions. Ultimately, such interventions may be a critical pathway to impede the intergenerational transmission of poor health from mothers to children and address disparate maternal outcomes by decreasing the risk of cardiovascular-related morbidity and mortality.

This study used the publicly available data from the Pregnancy Risk Assessment and Monitoring System (PRAMS), a population-based surveillance system that is administered by the Centers for Disease Control and Prevention (CDC) in collaboration with state, regional, and local health departments (17). PRAMS is focused on health indicators immediately before, during, and after pregnancy. Additional data about SDOH are either directly available or can be estimated from other questions that are a part of the core PRAMS questionnaire. PRAMS provides high national participation, covering 81% of all US live births. Further, PRAMS data are connected to birth records, and responses are collected within 3 to 6 months after a live birth. Thus, this measure is more reliable than other survey instruments that are often retrospectively administered postpregnancy.

Using the PRAMS data set, we sought to 1) identify the association between individual, interpersonal, and community-level SDOH with CVH in childbearing-age women and 2) explore the extent to which the association between SDOH and CVH varies by race and ethnicity.

## Methods

This cross-sectional study used data from Phase 8 (2016–2020) of the PRAMS data set. The PRAMS questionnaire consists of 2 parts: core questions (18) that are common across all participating sites (49 states and Puerto Rico) and standard questions (19), which vary to meet state-specific policy needs. The aim of PRAMS is to capture a snapshot of women’s health-related experiences and behaviors in the perinatal period. Using birth certificate records, women who had a recent live birth are sampled within 3 to 6 months either via telephone or mail to complete the PRAMS questionnaire. The complex survey design adopted by PRAMS uses sample weights for sample design, nonresponse, and noncoverage. States are included in the PRAMS annual data if they meet the minimum response rate threshold for the given period. The response rate threshold was set by CDC for 2016–2017 was 55%, while for 2018–2020 it was 50% (20). Therefore, states included in the survey vary from year to year. Detailed methods of PRAMS have been extensively documented (21) and reviewed by CDC as well as state review boards.

This study was reviewed by the University of Delaware Institutional Review Board and determined to be exempt.

### Measures

#### Cardiovascular health

To establish an person’s composite health score, we used a 3-level scoring model for each of the CVH variables collected in the PRAMS: body mass index (BMI) and history of diabetes, hypertension, and smoking. Scores of ideal (score = 2), intermediate (score = 1), and poor (score = 0) were respectively assigned to the person’s status for BMI (normal = 2, overweight = 1, obese or underweight = 0), diabetes (no history = 2, history of gestational diabetes = 1, history of diabetes mellitus = 0), hypertension (no history = 2, history of gestational hypertension = 1, history of chronic hypertension = 0), and smoking (never smoker = 2, previous smoker that quit = 1, current smoker = 0). Cases coded with both gestational and chronic hypertension reflected gestational hypertension that persisted and was reclassified as chronic; a similar process was applied to gestational diabetes that persisted and was reclassified as diabetes mellitus.

Women with 2 or more existing or previous health concerns (composite score of 0 to 6) were considered to have poor CVH, whereas those with 1 or no concerns (composite score of 7 or 8) were considered to have ideal CVH. We chose this binary classification because the small sample sizes in intermediate categories did not allow for stable estimates, particularly in subgroup analyses by race and ethnicity and SDOH. Collapsing into 2 categories thus maximized statistical power and allowed us to focus on the contrast between women at lowest risk and those with multiple concurrent risk factors.

#### Social determinants of health

The numbers presented below are coding categories, not scoring for SDOH. For the individual level SDOH, we used maternal age at the time of giving birth (1 = aged <18 years; 2 = aged 18–34 years; 3 = aged ≥35 years) and drinking status (0 = no history, 1 = history). Interpersonal level SDOH included marital status (0 = married, 1 = unmarried), birth order for the recorded pregnancy reflecting maternal parity (first, second, or third child), breast feeding history (0 = yes, 1 = no), history of depression diagnosis (0 = no current or past diagnosis, 1 = current or past depression diagnosis) and history of abuse by spouse or family (0 = no, 1 = yes). Breastfeeding was included as an interpersonal SDOH because, although measured postpartum in PRAMS, it reflects maternal intention, support, and resource access during pregnancy in the context of this cross-sectional study. Birth order and depression were included as interpersonal SDOH because both reflect the family and social contexts that shape maternal resources, support, and stress. The community-level SDOH in our data set were rural residency (0 = urban, 1 = rural), education (0 = high school diploma and higher, 1 = less than a high school diploma), work status (0 = working, 1 = nonworking), insurance status (0 = private, 1 = government, 2 = none), poverty level as determined by comparing the family income to the federal poverty level (FPL) (0 = above FPL, 1 = below FPL), and food security determined by use of the Supplemental Nutrition Program for Women, Infants, and Children (WIC) and other support (0 = food secure, 1 = food insecure). While WIC participation may improve food stability among low-income women, reliance on such support reflects reduced self-sufficiency and thus vulnerability in food security. The survey period (2016–2020) was used as a covariate to control for changes over the study period. We explored maternal race and ethnicity as an effect modifier between SDOH and CVH composite score. We grouped participants into 4 racial and ethnic categories: 1 = non-Hispanic White, 2 = non-Hispanic Black, 3 = Hispanic, and 4 = other, a catch-all category encompassing all remaining subgroups as defined in PRAMS.

### Statistical analysis

All analyses were conducted by using SAS statistical software (version 9.4) (SAS Institute) between September 2022 and February 2023, using survey procedures suitable for the complex survey design of PRAMS.

The initial data set of 206,080 participants was reduced to 205,513 participants who had complete CVH data. Some participants had missing information for variables due to survey item nonresponse and unavailable information in the birth records. Analysis of missing values showed no statistically significant patterns and hence the missingness was assumed to be at random. We used hot deck imputation procedures (22) to impute missing values in our exposure variables. Hot deck imputation handles missing observations by replacing each of them with an observed response from a similar observation. This study used age, smoking status, insurance status, and the survey year to establish similarity, which resulted in successful imputation of all missing values. All adjusted models included maternal age and survey year as covariates to control for demographic and temporal variation.

Weighted frequencies and percentages were calculated for each variable of interest. Stepwise multivariate logistic regression models were developed to assess the association between SDOH and CVH using an α level of .05. The process started with the development of bivariate models and successive adding of additional factors. Separate models for each of the individual, interpersonal, and community level SDOH were also tested. Product terms for race and ethnicity with all SDOH were assessed for interaction. All product terms were found to be significant at *P* < .05. Therefore, a stratified analysis was conducted for each racial subgroup.

## Results

Of the total sample of pregnant women (n = 205,513), more than half (57.2%) were non-Hispanic White, 15.1% were non-Hispanic Black, 18.3% were Hispanic, and 9.4% identified as other race and ethnicity (Table 1). The women in the study predominantly had poor CVH status (53.8%, n = 110,580). 

**Table 1:** Descriptive Statistics of Pregnant US Women Who Participated in the Phase 8 (2016–2020) Survey, by Cardiovascular Health (CVH) Status (N = 205,513), Pregnancy Risk Assessment and Monitoring System, 2016–2020

Factor	Overall sample	CVH status^a^^,^ b, n (weighted %)	
		Ideal (n = 94,933)	Poor (n = 110,580)
**Individual Factors**			
**Maternal age, y**			
<18	9,480 (4.3)	4,653 (50.7)	4,827 (49.3)^c^
18–34	157,331 (77.0)	73,062 (49.6)	84,269 (50.4)
≥35	38,702 (18.4)	17,218 (48.2)	21,484 (51.8)
**Maternal race and ethnicity**			
Non-Hispanic White	97,213 (57.2)	47,679 (51.1)	49,534 (48.9)^c^
Non-Hispanic Black	37,232 (15.1)	13,957 (39.8)	23,275 (60.2)
Hispanic	37,237 (18.3)	18,027 (50.8)	19,210 (49.2)
Other	33,831 (9.4)	15,270 (51.8)	18,561 (48.2)
**Body mass index group**			
Underweight	7,046 (3.2)	0	7,046 (100)^c^
Normal weight	88,224 (43.9)	64,357 (76.2)	23,867 (23.8)
Overweight	53,434 (26.3)	30,576 (60.6)	22,858 (39.4)
Obese	56,809 (26.5)	0	56,809 (100)
**Drinking history**			
No	72,593 (33.6)	33,558 (49.4)	39,035 (50.6)^c^
Yes	132,920 (66.4)	61,375 (49.4)	71,545 (50.6)
**Ever smoker**			
No	77,058 (38.5)	41,787 (56.7)	35,271 (43.3)^c^
Yes	128,455 (61.5)	53,146 (44.8)	75,309 (55.2)
**Diabetes**			
No	177,267 (87.2)	94,653 (56.5)	82,614 (43.5)^c^
Yes	28,246 (12.8)	280 (1.17)	27,966 (98.8)
**Hypertension**			
No	161,579 (81.7)	93,985 (60.0)	67,594 (40.0)^c^
Yes	43,934 (18.3)	948 (2.4)	42,986 (97.6)
**Interpersonal Factors**			
**Marital status**			
Unmarried	125,605 (63.5)	65,073 (54.7)	60,532 (45.3)^c^
Married	79,908 (36.5)	29,860 (40.1)	50,048 (59.9)
**Birth order of recorded pregnancy**			
First child	201,872 (99.1)	93,570 (49.5)	108,302 (50.5)^c^
Second child	3,575 (0.90)	1,343 (38.3)	2,232 (61.7)
Third child	66 (0.01)	20 (29.6)	46 (70.4)
**History of breast feeding **			
Yes	169,307 (84.0)	82,860 (51.9)	86,447 (48.1)^c^
No	36,206 (16.0)	12,073 (36.2)	24,133 (63.8)
**History of abuse **			
No	195,283 (95.6)	91,839 (50.2)	103,444 (49.8)^c^
Yes	10,230 (4.35)	3,094 (32.6)	7,136 (67.4)
**History of depression **			
No	107,418 (54.7)	55,512 (54.8)	51,906 (45.2)^c^
Yes	98,095 (45.3)	39,421 (42.8)	58,674 (57.2)
**Community Factors**			
**Rural residence**			
No	142,913 (73.7)	66,852 (50.4)	76,061 (49.6)^c^
Yes	62,600 (26.3)	28,081 (46.5)	34,519 (53.5)
**Education level**			
High school diploma or more	180,322 (88.1)	85,293 (50.5)	95,029 (49.5)^c^
Less than high school diploma	25,191 (11.9)	9,640 (41.4)	15,551 (58.6)
**Working status**			
No	115,814 (55.1)	51,409 (47.5)	64,405 (52.5)^c^
Yes	89,699 (44.9)	43,524 (51.7)	46,175 (48.3)
**Type of insurance**			
None	26,799 (14.5)	11,503 (45.9)	15,296 (54.1)^c^
Government	64,039 (26.3)	23,053 (38.7)	40,986 (61.3)
Private	114,675 (59.2)	60,377 (55.0)	54,298 (45.0)
**Living below federal poverty level**			
No	156,794 (78.3)	77,342 (52.4)	79,452 (47.6)^c^
Yes	48,719 (21.7)	17,591 (38.5)	31,128 (61.5)
**Food secure**			
No	127,017 (64.6)	65,438 (54.6)	61,579 (45.4)^c^
Yes	78,496 (35.4)	29,495 (39.9)	49,001 (60.1)

a CVH status as identified by CVH score: ideal (score, 7 or 8) or poor (score, 0–6).

b Rao-Scott χ^2^ test was used to examine whether overall differences in proportion of ideal versus poor CVH status are different between groups.

c Significant at *P* < .05.

The association of SDOH with CVH was moderated by maternal race and ethnicity, and the results were significantly different across the racial and ethnic subgroups (Table 2). Overall, compared with the reference group, older maternal age, history of drinking, unmarried status, higher number of pregnancies, lack of breastfeeding, history of abuse and depression, rural residency, lower education, lack of private insurance, poverty, and food insecurity were associated with higher adjusted odds of poor CVH. Odds of poor CVH increased with older maternal age, and these results were consistent across racial and ethnic subgroups, albeit at different magnitudes. Compared with other racial and ethnic subgroups, non-Hispanic White women aged 18 to 34 years had higher odds of poor CVH (AOR = 1.92; 95% CI, 1.69–2.19), whereas non-Hispanic Black women aged 35 years or older had the highest odds (AOR = 2.86; 95% CI, 2.36–3.46). White women reporting a third birth had the highest odds of poor CVH (AOR = 4.06; 95% CI, 1.51–10.92) compared with all other races and ethnicities reporting a second or third birth. In general, more disadvantaged SDOH status increased odds of poor CVH across all racial and ethnic subgroups, and non-Hispanic White women had the highest adjusted odds overall. Disadvantaged SDOH were associated with poor CVH, including rural residence (OR = 1.15), food insecurity (OR = 1.36), abuse history (OR = 1.43), depression history (OR = 1.52), living at or below the federal poverty line (OR = 1.23), single marital status (OR = 1.38), less than high school diploma (OR = 1.13) and having government insurance (OR = 1.31) (Table 2).

**Table 2:** Odds of Pregnant Women Having Poor Cardiovascular Health Based on Social Determinants of Health, by Maternal Race and Ethnicity, Pregnancy Risk Assessment and Monitoring System, 2016–2020

Factor	Overall	Maternal race and ethnicity			
		Non-Hispanic White	Non-Hispanic Black	Hispanic	Other^a^
	Adjusted odds ratio (95% CI)				
**Individual factors**					
**Maternal age, y**					
<18	1 [Reference]	1 [Reference]	1 [Reference]	1 [Reference]	1 [Reference]
18–34	1.72 (1.58–1.86)	1.92 (1.69–2.19)	1.67 (1.42–1.98)	1.54 (1.32–1.78)	1.48 (1.14–1.93)
≥35	2.17 (1.99–2.37)	2.21 (1.93–2.54)	2.86 (2.36–3.46)	2.14 (1.81–2.53)	1.95 (1.47–2.58)
**Drinking history**					
No	1 [Reference]	1 [Reference]	1 [Reference]	1 [Reference]	1 [Reference]
Yes	1.16 (1.12–1.20)	1.11 (1.06–1.17)	1.40 (1.30–1.52)	1.12 (1.04–1.21)	1.18 (1.09–1.29)
**Interpersonal Factors**					
**Marital status**					
Married	1 [Reference]	1 [Reference]	1 [Reference]	1 [Reference]	1 [Reference]
Unmarried	1.38 (1.33–1.43)	1.51 (1.43–1.59)	1.15 (1.05–1.25)	1.13 (1.05–1.22)	1.43 (1.28–1.59)
**Birth order of recorded pregnancy**					
First child	1 [Reference]	1 [Reference]	1 [Reference]	1 [Reference]	1 [Reference]
Second child	1.55 (1.36–1.76)	1.56 (1.33–1.84)	1.38 (0.99–1.91)	1.55 (1.13–2.14)	2.18 (1.38–3.46)
Third child	2.58 (1.16–5.76)	4.06 (1.51–10.92)	0.90 (0.22–3.66)	1.85 (0.21–16.0)	1.89 (0.24–15.16)
**History of breastfeeding**					
Yes	1 [Reference]	1 [Reference]	1 [Reference]	1 [Reference]	1 [Reference]
No	1.57 (1.51–1.63)	1.64 (1.55–1.74)	1.37 (1.26–1.50)	1.35 (1.21–1.50)	1.42 (1.25–1.61)
**History of abuse**					
No	1 [Reference]	1 [Reference]	1 [Reference]	1 [Reference]	1 [Reference]
Yes	1.43 (1.33–1.55)	1.58 (1.41–1.78)	1.12 (0.96–1.31)	1.25 (1.06–1.47)	1.35 (1.11–1.66)
**History of depression**					
No	1 [Reference]	1 [Reference]	1 [Reference]	1 [Reference]	1 [Reference]
Yes	1.52 (1.47–1.57)	1.56 (1.49–1.62)	1.43 (1.32–1.55)	1.42 (1.32–1.54)	1.50 (1.37–1.64)
**Community Factors**					
**Rural residence**					
No	1 [Reference]	1 [Reference]	1 [Reference]	1 [Reference]	1 [Reference]
Yes	1.15 (1.12–1.19)	1.15 (1.10–1.20)	1.00 (0.90–1.09)	1.12 (1.03–1.21)	1.20 (1.09–1.31)
**Education level**					
High school diploma or more	1 [Reference]	1 [Reference]	1 [Reference]	1 [Reference]	1 [Reference]
Less than high school diploma	1.13 (1.07–1.19)	1.35 (1.23–1.48)	1.20 (1.06–1.36)	1.11 (1.01–1.21)	1.21 (1.04–1.40)
**Working status**					
Yes	1 [Reference]	1 [Reference]	1 [Reference]	1 [Reference]	1 [Reference]
No	0.96 (0.93–0.99)	0.96 (0.92–1.00)	0.90 (0.83–0.97)	0.97 (0.90–1.05)	0.98 (0.90–1.07)
**Type of insurance**					
Private	1 [Reference]	1 [Reference]	1 [Reference]	1 [Reference]	1 [Reference]
Government	1.31 (1.26–1.37)	1.35 (1.26–1.43)	1.07 (0.98–1.18)	1.11 (1.01–1.22)	1.46 (1.31–1.63)
None	1.16 (1.11–1.22)	1.36 (1.26–1.47)	1.05 (0.93–1.20)	1.00 (0.91–1.10)	1.22 (1.03–1.44)
**Living below federal poverty level**					
No	1 [Reference]	1 [Reference]	1 [Reference]	1 [Reference]	1 [Reference]
Yes	1.23 (1.18–1.28)	1.33 (1.24–1.42)	1.19 (1.09–1.30)	1.19 (1.10–1.29)	1.14 (1.01–1.28)
**Food secure**					
Yes	1 [Reference]	1 [Reference]	1 [Reference]	1 [Reference]	1 [Reference]
No	1.36 (1.31–1.41)	1.56 (1.48–1.65)	1.20 (1.11–1.30)	1.15 (1.06–1.24)	1.22 (1.10–1.35)

a Other includes American Indian, Hawaiian, Alaska Native, Chinese, Japanese, Filipino, other Asian, mixed race, and other non-White.

## Discussion

We found a significant association between adverse CVH outcomes and lower or worse SDOH characteristics at the individual, interpersonal, and community levels. We also observed that pregnant women of all racial and ethnic backgrounds were susceptible to adverse CVH outcomes at lower SDOH levels, with a pronounced increase in odds of adverse outcomes among White women across all studied SDOH factors. Our findings can assist in the development of interventions for improving CVH outcomes in high-risk women of childbearing age.

Our sample showed a higher prevalence of diabetes and lower prevalence of hypertension compared with the general US population (23,24), and most of our findings align with existing literature. These findings included the worsening gestational CVH with increasing maternal age (25), the association between drinking and adverse CVH (26), the protective effects of breastfeeding (27), and the detrimental effect of interpersonal trauma (eg, child abuse) and depression on CVH (28,29). Additionally, consistent with prior research, we found associations between CVH disparities and rural populations (30), low maternal education (31), lack of health insurance, poverty, and food insecurity (32). Considering the interconnected and multilevel SDOH linked to CVH, adopting an integrated approach to address these SDOH across various levels and tailoring it to specific subgroups (eg, older gestational age, single parents) is essential to maximize the impact of CVH interventions in the community.

We found that non-Hispanic White women with disadvantaged SDOH factors, such as being unmarried, lack of breast feeding, having a history of abuse, lack of health insurance, low education levels, and food insecurity, had the greatest odds of adverse CVH outcomes compared with women in other racial and ethnic groups. Although this pattern could be interpreted that non-Hispanic White women have more access to health-promoting SDOH resources, this interpretation must be made with caution given the cross-sectional nature of the data; evidence on the racial and ethnic differences in the association between SDOH and CVH is limited. Nevertheless, our results align with a nationally representative study involving adolescents aged 12 to 19 years. The association between SDOH and CVH was most pronounced among non-Hispanic White adolescents, moderately significant among Mexican-American adolescents, but not significant among non-Hispanic Black adolescents (33). The unequal associations between SDOH and CVH may be related to the marginalization-related diminished return (34,35), wherein the protective benefits of social and economic resources are less pronounced among racial and ethnic minority groups due to structural barriers. Structural factors such as segregation, racism, and discrimination may have diminished the influence of other SDOH (36) among minority groups while amplifying observed associations among non-Hispanic White women. Although not a primary objective of this study, prior research has consistently shown that minority racial and ethnic groups experience a disproportionate burden of adverse SDOH, which likely contributes to disparities in maternal CVH. Future research is needed to elucidate racial disparities on the impact of SDOH on CVH to inform public health efforts to reduce CVH disparities.

Our study has several strengths. We used the core question responses in the PRAMS data set to increase generalizability of findings across the wider US population and capitalized on the strengths of PRAMS, which mitigates the potential for recall bias when relying on long-term recollection by promptly collecting data immediately following pregnancy. PRAMS, weighted for national representativeness, is linked with birth records, thereby enhancing objectivity in variables such as hypertension, BMI, and diabetes. However, certain limitations are inherent in the PRAMS data set. The system encompasses limited information on SDOH, a deficiency recently recognized by CDC as an ongoing challenge (37). The most recent version of the PRAMS questionnaire (Phase 9) has added topical areas for SDOH (38). In addition, the operationalization of CVH in this study was limited to 4 components available in PRAMS (BMI, smoking, diabetes, and hypertension). Other critical components of the American Heart Association’s Life’s Essential 8 (eg, diet, physical activity, sleep health, and cholesterol) were not collected in the PRAMS core questionnaire and therefore could not be included. This restricted operationalization may underestimate the prevalence of poor CVH and could misclassify women who have poor CVH due to unmeasured behaviors or biological risk factors. Consequently, our findings may provide a conservative estimate of the true burden of poor CVH among pregnant women. The indicators used in this study did not account for the physiological changes that happen during pregnancy that can impact CVH (39). Furthermore, some SDOH and behavioral measures, such as smoking, depression, and abuse history, relied on maternal self-report. Although PRAMS reduces long recall periods by administering surveys within 3 to 6 months postpartum, these measures remain subject to recall bias and potential misclassification. Such biases may have led to underestimation of associations between SDOH and CVH. Residual confounding is possible because some covariates such as prepregnancy health status, physical activity level, and neighborhood-level characteristics were not available in PRAMS. These unmeasured variables may partially explain observed associations.

The small sample size within the third pregnancy subgroup may have introduced a potential limitation to the reliability of our assertions for this specific subgroup. To mitigate data gaps, statistical imputation was applied to address missing values in many records, enhancing the completeness and robustness of our analysis. These considerations underscore the need for a nuanced interpretation of our findings and emphasize the ongoing efforts to refine data collection methods, particularly concerning SDOH in pregnancy research. Because of the cross-sectional design, causal inferences cannot be made; our findings reflect associations between SDOH and CVH during pregnancy.

Addressing SDOH necessitates social and environmental policy changes that inform health interventions. Providers should consider these factors in their care strategies and advocate for SDOH policy changes that can support the development of health behavior interventions, longitudinal study designs, and the identification of underlying mechanisms through mediation analysis. All pregnant women in the US are vulnerable to CVH risks, irrespective of race. Assessing how CVH risks are associated with race and ethnicity and socioeconomic disadvantage separately highlights the need to reconsider an overreliance on the grouping of these factors due to their frequent coexistence.

The effect of SDOH on health outcomes is applicable to women of all races and ethnicities. The effect on pregnant women is noteworthy, as the adverse outcomes do not stop with the mother and tend to have a transgenerational effect.
